# A Handbook of Dental Pathology for Students and Practitioners

**Published:** 1889-10

**Authors:** 


A HANDBOOK OF DENTAL PATHOLOGY FOR STUDENTS AND PRACTITIONERS.—By Albert N. Bodgett, M. D., late Professor of Pathology in Boston Dental College. Published by P. Blakiston, Son & Co., Philadelphia, 1888.

Part 1. Describes the anatomy of the jaws, salivary glands and saliva, and their pathological conditions, structure of the teeth, development, primary dentition and eruption of the teeth, absorption of deciduous teeth, and secondary dentition and the development and eruption of the permanent teeth.

Part 2. Is devoted to general pathology of the teeth.

Part 3. Describes the relation of the digestive organs to diseases of the mouth and teeth, the effects of bacteria, defective embryonic development of maxillary structures, and histological development of the teeth.

Part 4. Relates to pathological conditions, associated with second dentition; anomalies, formative defects, deficiency of teeth, variations in size, fusion of teeth, rachitis, inflammatory affections, gangrene of pulp, diseases of the teeth and processes.

Part 5. Describes the courses, &c., of acute inflammation of hard structures and of the teeth, pathological and malignant growths, hypertrophy, carcinoma, enchondroma and sarcoma.

This treatise is written in a clear and satisfactory style, and imparts knowledge of the utmost importance to the dental student and practitioner. The illustrations are numerous and well executed, and add greatly to the value of the work.

